# Higher Adherence to the Mediterranean Diet Is Associated with a Lower Risk of Steatotic, Alcohol-Related, and Metabolic Dysfunction-Associated Steatotic Liver Disease: A Retrospective Analysis

**DOI:** 10.3390/nu16203551

**Published:** 2024-10-19

**Authors:** Ji Yae Lee, Sue Kim, Yaeji Lee, Yu-Jin Kwon, Ji-Won Lee

**Affiliations:** 1 Department of Family Medicine, Severance Hospital, Yonsei University College of Medicine, Seoul 03722, Republic of Korea; leelee@yuhs.ac; 2 International Health Care Center, Severance Hospital, Yonsei University Health System, Seoul 03722, Republic of Korea; ladysueya@gmail.com; 3 Department of Biostatistics and Computing, Yonsei University, Seoul 03722, Republic of Korea; ysbiostat@yuhs.ac; 4 Department of Family Medicine, Yongin Severance Hospital, Yonsei University College of Medicine, Yongin 16995, Republic of Korea; 5 Department of Family Medicine, Severance Hospital, Yonsei University College of Medicine, Institute for Innovation in Digital Healthcare, Yonsei University, Yonsei-ro 50-1, Seodaemun-gu, Seoul 03722, Republic of Korea

**Keywords:** Mediterranean diet adherence, steatotic liver disease, metabolic dysfunction-associated steatotic liver disease, alcohol-related liver disease

## Abstract

Background and Aims: Metabolic liver disease is associated with obesity, insulin resistance, cardiovascular disease, and metabolic disorders. A Mediterranean diet (MD), known for its anti-inflammatory and antioxidant properties, is effective in managing various chronic diseases, including liver diseases. This study aimed to explore the influence of adherence to the MD on the risk of chronic metabolic diseases, including steatotic liver disease (SLD), metabolic dysfunction-associated steatotic liver disease (MASLD), and alcohol-related liver diseases (ALDs). Methods: This retrospective cohort study analyzed 5395 individuals from a single center between 2020 and 2022, grouped by adherence to the MD using the Korean Mediterranean Diet Adherence Score (K-MEDAS). MASLD score, ALD, and cardiovascular risk factors were also assessed. Statistical analyses were performed using 1:1 exact matching and multiple regression to compare the less adherent (K-MEDAS 0–7) and highly adherent (K-MEDAS 8–13) groups. Results: Adjusting for confounding variables, high adherence to the MD was significantly associated with lower rates of SLD (odds ratio [OR] 0.818, 95% confidence interval [CI] 0.700–0.957, *p* = 0.012), MASLD (OR 0.839, 95% CI 0.714–0.986, *p* = 0.033), and ALD (OR 0.677, 95% CI 0.671–0.683, *p* < 0.001). Post-propensity score matching analysis revealed that the highly adherent group exhibited significantly lower triglyceride levels, triglyceride and glucose index, atherogenic Index of Plasma, and Framingham risk scores than the less adherent group. Conclusions: Good adherence to the MD considerably reduces the risk of SLD, MASLD, and ALD, underscoring its protective effects and potential to prevent metabolic liver diseases and their complications.

## 1. Introduction

A recent shift in liver disease terminology has replaced nonalcoholic fatty liver disease (NAFLD) and nonalcoholic steatohepatitis (NASH) with new terms: metabolic dysfunction-associated fatty liver disease (MAFLD) and metabolic dysfunction-associated steatotic liver disease (MASLD). This change better reflects the multifactorial and metabolic origins of these conditions [1]. Steatotic liver disease (SLD) encompasses conditions that lead to hepatic steatosis, such as MASLD and alcohol-related liver disease (ALD). A new category, metabolic dysfunction and alcohol-related liver disease (MetALD), has been introduced for cases where MASLD occurs with alcohol consumption above the NAFLD, which limits daily intake to <20 g/30 g for females and males, respectively, but below 50 g/day for women and 60 g/day for men. This category reflects the combined effects of alcohol consumption and metabolic risks, acknowledging the spectrum of the disease rather than considering these conditions separately [2]. The underlying mechanisms of liver diseases, such as insulin resistance and systemic inflammation, highlight the importance of metabolic health in the progression of liver diseases [3]. Untreated, these conditions can lead to cirrhosis, liver failure, or hepatocellular carcinoma (HCC), underscoring the need for early detection and intervention [3].

Cardiovascular risks are markedly increased in patients with metabolic liver diseases, sharing mutual risk factors such as obesity and metabolic syndrome [4]. The American Heart Association’s 2011 projections estimate that by 2030, over 40% of the U.S. population, exceeding 100 million people, will be affected by cardiovascular diseases (CVDs). These conditions have tremendous economic repercussions. The financial burden of CVD in 2016 was USD 555 billion, with costs expected to surge to USD 1.1 trillion by 2035 [5].

Concurrently, the Mediterranean diet (MD), notable for its rich intake of plant-derived foods, such as vegetables, fruits, legumes, nuts, whole grains, and olive oil, and minimal reliance on animal products, has been acknowledged for its positive health impacts [6,7]. Previous studies have revealed that MD could reduce the risk of chronic diseases such as CVD, stroke, type 2 diabetes mellitus (T2DM), and certain cancers, as well as provide benefits related to weight management [8,9,10,11,12,13]. While the precise pathways through which the MD confers these advantages are not fully known, current research highlights several key benefits: it lowers lipid levels, shields against inflammation and oxidative damage, affects hormonal and growth factor pathways that can lead to cancer, and promotes the generation of beneficial metabolites through gut microbiota interactions, all of which are crucial for metabolic health [14]. Given that there is currently no approved pharmacological treatment for MASLD [15], finding alternative approaches to managing metabolic liver disease is crucial. The most recent MASLD guidelines, published by the European Society for Clinical Nutrition and Metabolism, recommend the MD to reduce hepatic steatosis and improve insulin sensitivity [16].

Considering these interconnections, our study aimed to explore the association of adherence to the MD with chronic metabolic diseases, including SLD, MASLD, and ALD.

## 2. Materials and Methods

### 2.1. Study Design and Data Collection

**Figure 1:**
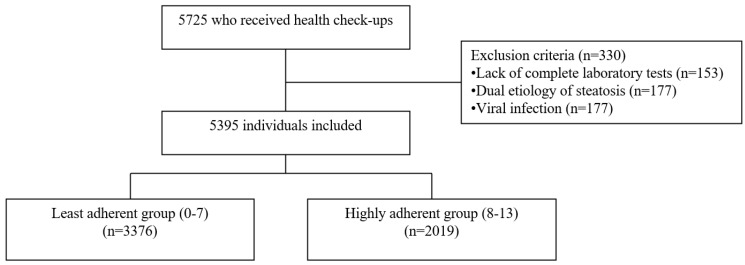
Flow chart of participants. This chart shows the inclusion flowchart of the study participants. After exclusion, participants were divided into two groups according to their adherence to the Mediterranean diet score: the least adherent (K-MEDAS 0–7) and highly adherent (K-MEDAS 8–13). K-MEDAS: Korean Mediterranean Diet Adherence Score [17].

This retrospective cohort study analyzed 5725 individuals aged 14–88 years who attended the Health Checkup Center at Yongin Severance Hospital between March 2020 and September 2022. From the initial cohort, 330 subjects were excluded due to the following predetermined criteria: 153 with incomplete laboratory tests and 177 with a dual etiology of steatosis, which included cases of viral infection. The remaining 5395 individuals were stratified into two groups according to their adherence to the MD, with 3376 classified as ‘least adherent’ and 2019 classified as ‘highly adherent’ (Figure 1). These groupings facilitated subsequent analyses examining the association of diet adherence with the prevalence of SLD, MASLD, and ALD, as well as related cardiovascular risk factors. This study was approved by the Institutional Review Board of Yongin Severance Hospital (IRB No. 9-2021-0099) and was performed in compliance with the Declaration of Helsinki.

Data on adherence to the MD were gathered using the Korean Mediterranean Diet Adherence Score (K-MEDAS), a 14-item scoring system reflecting the degree of adherence to the MD patterns in the Korean population, developed by Lee et al. [17]. K-MEDAS accurately represents adherence to the MD, reflecting the Korean diet, which considerably correlates with the Food Frequency Questionnaire (FFQ), a 106-item questionnaire developed by the Korean Genome and Epidemiology Study [18]. K-MEDAS points were assigned for using perilla or olive oil as the primary cooking fat (Q1) and preferring white meat over red meat (Q13). Additional points were earned for consuming at least three teaspoons of perilla or olive oil daily (Q2), eating two or more vegetable servings per day (Q3), consuming one or more pieces of fruit each day (Q4), and limiting red meat and sausages to less than one serving daily (Q5). Further points were given for consuming less than one serving per day of butter, margarine, or cream (Q6), less than one sugar-sweetened beverage per day (Q7), seven or more servings of wine per week (Q8), three or more servings weekly of beans or tofu (Q9), and three or more servings per week of fish or seafood (Q10). Additionally, points were given for consuming sweet bread (excluding whole wheat), cakes, and cookies fewer than three times per week (Q11), eating nuts three times or more per week (Q12), and consuming whole grains at least three times per week (Q14). Each unmet criterion resulted in a zero score, with the total possible score ranging from 0 to 14, where a higher score indicates greater adherence to the MD [17]. 

Participants were categorized into two groups according to the median of the K-MEDAS: those scoring between 0–7 were considered less adherent, while those scoring 8–13 were classified as highly adherent to the MD. Lifestyle factors were assessed using self-administered questionnaires. Categories of smoking status included never-smokers, former smokers, and active smokers. Alcohol use was identified based on current drinking habits. Physical activity level was evaluated using the International Physical Activity Questionnaire and quantified in terms of the metabolic equivalent of task (MET) units. A history of hypertension, T2DM, dyslipidemia, cardiovascular risk, and cancer was self-reported by the participants.

### 2.2. Anthropometric and Laboratory Measurements

Body mass index (BMI) was determined by dividing weight in kilograms by the square of height in meters, a widely used measure of obesity endorsed by the World Health Organization (WHO) [19]. Waist circumference (WC) was also measured, according to WHO guidelines, at the midpoint between the iliac crest and costal margins [19]. Blood pressure readings were obtained twice after the individual had been seated and resting for 5 min [20]. For blood sample analysis, which was collected after an overnight fast, levels of fasting serum glucose, total cholesterol, triglycerides (TG), high-density lipoprotein cholesterol (HDL-C), low-density lipoprotein cholesterol (LDL-C), aspartate aminotransferase (AST), alanine aminotransferase (ALT), γ-glutamyl transferase (GGT), and C-reactive protein (CRP) were measured using the ADVIA 1800 Clinical Chemistry System by Siemens Healthcare Diagnostic, Inc., Forchheim, Germany. 

### 2.3. Definition of SLD, MASLD, and ALD

SLD was diagnosed based on one of the following criteria: (1) hepatic steatosis confirmed by liver biopsy or (2) ultrasound imaging showing steatosis [2]. 

MASLD diagnosis required the presence of SLD identified by ultrasound imaging, along with any cardiometabolic risk factors and a self-reported alcohol intake of less than 20 g/day for women or 30 g/day for men. The cardiometabolic criteria for adults include at least one of the following: a BMI ≥ 25 kg/m^2^ (or ≥23 kg/m^2^ for Asian populations) or a WC that exceeds 94 cm for men and 80 cm for women [21]; fasting serum glucose ≥ 100 mg/dL (5.6 mmol/L), 2 h post-load glucose ≥ 140 mg/dL (7.8 mmol/L), HbA1c ≥ 5.7%, or the presence of T2DM or treatment for it; blood pressure ≥ 130/85 mmHg or the use of antihypertensive treatment; plasma TG ≥ 150 mg/dL (≥1.70 mmol/L) or receiving lipid-lowering treatment; and plasma HDL-C < 40 mg/dL (1.0 mmol/L) for men and <50 mg/dL (1.3 mmol/L) for women or lipid-lowering treatment [21].

ALD was diagnosed in patients with SLD who exhibited at least one cardiometabolic risk factor and reported consuming alcohol in excess of 50 g/day for women and 60 g/day for men [2].

### 2.4. Cardiovascular Risk Assessment

The Framingham score, a sex-specific algorithm used to estimate the 10-year cardiovascular risk of an individual according to the Canadian Cardiovascular Society, was used to assess cardiovascular risk [22]. The atherogenic index of plasma (AIP) score, calculated as log (TG/HDL-C), was also used to assess cardiovascular risk [23]. Additionally, the triglyceride and glucose (TyG) index, calculated using the formula: ln (fasting TG [mg/dL] × fasting plasma glucose [mg/dL]/2), served as a marker for insulin resistance and a predictive index for cardiovascular risk [24].

### 2.5. Statistical Analysis

Data are expressed as mean ± standard deviation (SD) or number (percentage). The characteristics of the adherent groups were compared using Student’s *t*-test for continuous variables and Pearson’s chi-square test for categorical variables. 

We employed 1:1 exact matching to minimize confounding by pairing subjects in the least adherent group with those in the highly adherent group who have identical values for age and sex. This method ensures that the least adherent and highly adherent groups are balanced in terms of key demographic characteristics, enabling more accurate and unbiased comparisons of outcomes. By controlling for age and sex, this approach reduces potential bias in the analysis and allows for a clearer assessment of the adherence effect on the outcome. Post-matching demographics of the adherent groups were compared using the generalized estimating equation (GEE) method to account for the characteristics of the matched data.

Multiple regression analyses using a generalized linear mixed model (GLMM) compared the less adherent and highly adherent groups associated with SLD, MASLD, and ALD. Four models were used in the analysis. Model 1 was unadjusted. Model 2 was adjusted for age, sex, and BMI. Model 3 included adjustments for the variables in Model 2 and exercise, smoking status, and alcohol habits. Model 4 included adjustments for the variables in Model 3, along with a history of hypertension, T2DM, dyslipidemia, CVD, and cancer. The results were reported as odds ratios (ORs) and 95% confidence intervals (CIs). Furthermore, a subgroup analysis was performed. A *p*-value less than 0.05 was considered statistically significant. All analyses were conducted using R software version 4.3.0 (R Foundation for Statistical Computing, Vienna, Austria, http://www.R-project.org/), accessed on 11 June 2024.

## 3. Results

Table 1 shows the clinical characteristics of participants before and after matching for age and sex. Prior to matching, the less adherent group had a mean age of 47.0 ± 13.0 years, considerably younger than the highly adherent group, which had a mean age of 55.5 ± 12.0 years. Additionally, the proportion of male participants was higher in the less adherent group (56.2%) compared to the highly adherent group (46.0%). 

The less adherent group engaged in less exercise, consumed more alcohol, and had a lower prevalence of hypertension, T2DM, dyslipidemia, CVDs, and cancer (all *p* < 0.05).

After applying 1:1 exact matching for age and sex, both groups had a mean age of approximately 53.8 ± 11.7 years, with an equal male percentage of 49.9%. Post-propensity score matching revealed that the less adherent group had a higher prevalence of current smokers (19.5%), less physical activity (58.0%), and greater alcohol consumption (46.2%) compared to the highly adherent group (all *p* < 0.05). Significant differences were also observed in TG, TyG index, AIP, GGT, SLD prevalence, and Framingham risk score (*p* = 0.046, 0.013, 0.046, 0.034, 0.024, and 0.009, respectively).

Table 2 presents the results of the multiple regression analyses, which examine the relationship between high and low adherence to the MD and conditions such as SLD, MASLD, and ALD. The analyses were performed on a matched dataset generated through exact matching. To account for the characteristics of this matched data, a generalized linear mixed model (GLMM) was used. Compared to the less adherent group, the ORs and 95% Cis for the highly adherent group were 0.842 (0.732–0.969) for SLD, 0.871 (0.756–1.004) for MASLD, and 0.686 (0.683–0.689) for ALD. After adjusting for sex, BMI, alcohol intake, smoking status, exercise, history of hypertension, T2DM, dyslipidemia, CVD, and cancer, the Ors and 95% Cis for the highly adherent group were 0.818 (0.700–0.957) for SLD, 0.839 (0.714–0.986) for MASLD, and 0.677 (0.671–0.683) for ALD, respectively.

**Figure 2:**
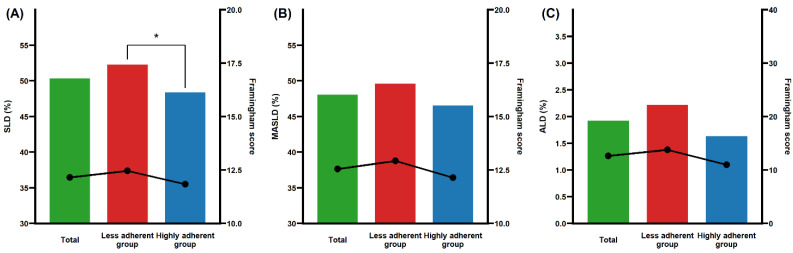
Prevalence of (**A**) SLD, (**B**) MASLD, and (**C**) ALD according to MD adherence. The bar graph illustrates the incidence of each metabolic liver condition in percentage across different levels of MD adherence: green bars represent the total participants, red bars represent the less adherent group, and blue bars represent the highly adherent group. The line graph overlays the Framingham score for each metabolic liver condition, also categorized by MD adherence. Significant values are indicated with an asterisk in the figure. MD, Mediterranean diet; SLD, steatotic liver disease; MASLD, metabolic dysfunction-associated steatotic liver disease; ALD, alcohol-related liver disease.

Figure 2 illustrates the prevalence of SLD, MASLD, ALD, and the Framingham score according to adherence to the MD. The bar graph represents the prevalence of metabolic liver conditions, while the line graph displays the Framingham score. The overall figure indicates a clear trend: both the prevalence of metabolic liver conditions and the Framingham score are lower in the highly adherent MD group compared to the less adherent population. A significant result is observed in SLD (Figure 2A), where the prevalence of SLD is notably higher in the less adherent group than in the highly adherent group.

**Figure 3:**
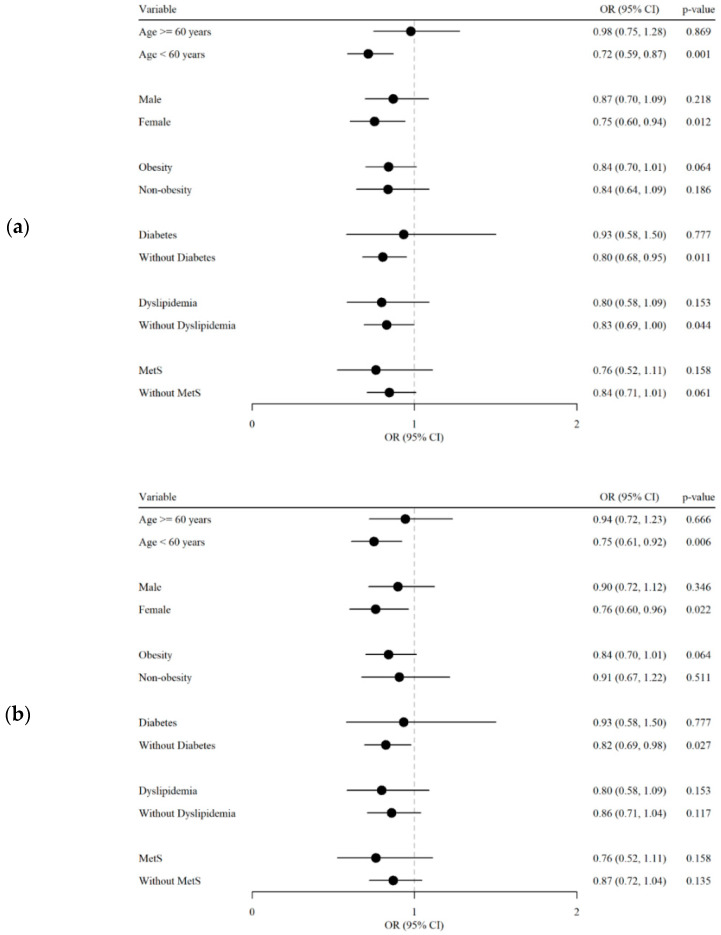
Subgroup analysis of metabolic liver diseases. (**a**) Forest plot for association between SLD and clinical & metabolic conditions. (**b**) Forest plot for association between MASLD and clinical & metabolic conditions. OR, odds ratio; CI, confidence interval. *p*-value by a generalized mixed model with a logit link.

Figure 3 presents a subgroup analysis of SLD and MASLD, focusing on the association between treatment and clinical outcomes across patient subtypes. In the SLD analysis (Figure 3a), individuals younger than 60 years showed significant benefit from high MD adherence (OR 0.72, 95% CI 0.59–0.87, *p* = 0.001), and females showed significantly better outcomes (OR 0.75, 95% CI 0.60–0.94, *p* = 0.012). Additionally, the absence of T2DM and dyslipidemia was associated with better outcomes (OR 0.80, 95% CI 0.68–0.95, *p* = 0.011; OR 0.83, 95% CI 0.69–1.00, *p* = 0.044). In the MASLD analysis (Figure 3b), younger individuals under 60 years benefited significantly from high MD adherence (OR 0.75, 95% CI 0.61–0.92, *p* = 0.006), and females showed more favorable results (OR 0.76, 95% CI 0.60–0.96, *p* = 0.022). Patients without T2DM had higher odds of developing MASLD (OR 0.82, 95% CI 0.69–0.98, *p* = 0.027). 

## 4. Discussion

### 4.1. Adherence to the Mediterranean Diet and Liver Disease

In this study, using the K-MEDAS questionnaire, we investigated the relationship between adherence to the MD and the prevalence of SLD, MASLD, and ALD in a Korean population. After total adjustment using a propensity score matching set including variables such as age and sex, high adherence to the MD was significantly associated with lower SLD, MASLD, and ALD rates. These findings are consistent with those of previous studies indicating the preventive effects of the MD on NAFLD [25]. Research conducted in Australia demonstrated that adherence to the MD for 6 weeks resulted in the reversal of NAFLD, as evidenced by a 39% reduction in intrahepatic lipids and a decrease in insulin resistance, which occurred independently of weight loss [26]. These results suggest that MD should be recommended for patients with NAFLD. Other studies have shown that greater adherence to MD in patients with NAFLD can lead to improvements in fibrosis, inflammatory markers, and various cardiovascular risk factors [27,28,29].

### 4.2. Potential Mechanisms

The precise relationship between the MD and liver diseases is not fully understood. One possible explanation is that the MD, rich in whole grains, fruits, and vegetables, can improve insulin sensitivity and regulate blood sugar levels, reducing the risk of insulin resistance, which is often linked to liver disease [25]. Additionally, the consumption of healthy fats, such as extra virgin olive oil, along with a balanced intake of complex carbohydrates, has been shown to decrease hepatic fat content. This is achieved by downregulating transcription factors involved in adipogenesis and lipogenesis, such as peroxisome proliferator-activated receptor and carbohydrate response element-binding protein [29,30,31]. Consistent with previous results, our study found that the group highly adherent to the MD had lower triglycerides and insulin resistance, as indicated by the TyG index. Another possible explanation is that MD is rich in compounds, such as polyphenols, vitamins, and other biomolecules that exhibit anti-inflammatory and antioxidant properties. Since inflammation and oxidative stress play crucial roles in the progression of both liver diseases and CVDs, dietary components targeting these processes could have beneficial effects on liver and vascular health [30]. NAFLD is considerably associated with a higher risk of cardiovascular events and other cardiac complications independent of traditional cardiovascular risk factors [31]. In our results, the highly adherent group had lower cardiovascular risk markers, such as AIP and Framingham Risk Score, than the less adherent group.

### 4.3. Subgroup Analysis

In a subgroup analysis, SLD and MASLD showed that high adherence to the MD significantly improved outcomes in individuals under 60 years of age, females, and patients without T2DM or dyslipidemia. Younger patients with higher basal metabolic rates and better physiological resilience may be more responsive to dietary interventions [32]. In line with our results, previous studies have also revealed notable sexual dimorphism in the prevalence and progression of SLD and MASLD [33,34]. Additionally, individuals without diabetes or dyslipidemia and with fewer metabolic disturbances may experience improved metabolic flexibility, making the benefits of the MD more pronounced [35,36]. Further research is required to elucidate the exact underlying mechanisms.

### 4.4. Study Limitations

Our study has some limitations. Primarily, its cross-sectional design restricts the ability to determine causal relationships. Secondly, the ALD group had a relatively smaller number of patients compared to other cohort studies, especially when the recruited participants were categorized into SLD, MASLD, and ALD groups. Third, the data were derived from a single center and self-reports, which limits generalization to the entire population, and misreporting cannot be completely ruled out. Future studies should address these limitations by including diverse ethnic groups and conducting multicenter studies to enhance the quality of the findings.

### 4.5. Study Strengths

Despite these limitations, our study offers several strengths. It is the first to investigate the relationship between MD adherence and the prevalence of SLD, MASLD, and ALD within a Korean population. Additionally, we diagnosed SLD using ultrasound imaging to detect steatosis, whereas most other studies have used indices calculated from laboratory findings. Ultrasonography, the recommended first-line imaging modality for diagnosing chronic liver disease and screening HCC, is relatively inexpensive, widely accessible, and radiation-free, making it suitable for population-based epidemiological studies [37]. It also shows moderate sensitivity with precise histological confirmation [38]. Furthermore, to minimize confounding effects, we employed exact 1:1 matching, aligning individuals from the least adherent group with those in the highly adherent group who shared the same age and sex. This matching process ensures that both groups are demographically comparable, allowing for more reliable and unbiased comparisons of their outcomes. By adjusting for these key demographic factors, our approach significantly reduces potential bias, enabling a clearer evaluation of how MD adherence influences the results.

## 5. Conclusions

Adherence to MD substantially reduces the risk of SLD, MASLD, and ALD, underscoring its protective effects and potential to prevent metabolic liver diseases and their complications. Future studies should examine the effects and mechanisms of MD adherence in a larger and more ethnically diverse cohort.

## Figures and Tables

**Table 1:** Clinical characteristics of study participants.

Characteristics	Total (*n* = 5395)	Before Matching			After Matching		
		Least Adherent Group (0–7)	Highly Adherent Group (8–13)	*p*	Least Adherent Group (0–7)	Highly Adherent Group (8–13)	*p*
		(*n* = 3376)	(*n* = 2019)		(*n* = 1716)	(*n* = 1716)	
Age, years	50.2 ± 13.3	47.0 ± 13.0	55.5 ± 12.0	<0.001	53.8 ± 11.7	53.8 ± 11.7	>0.999
Male Sex, *n* (%)	2825 (52.4%)	1897 (56.2%)	928 (46.0%)	<0.001	857 (49.9%)	857 (49.9%)	>0.999
BMI, kg/m^2^	24.2 ± 3.5	24.2 ± 3.6	24.3 ± 3.3	0.743	24.2 ± 3.4	24.3 ± 3.3	0.435
SBP, mmHg	122.9 ± 14.1	122.2 ± 14.2	124.3 ± 13.9	<0.001	124.2 ± 13.9	123.7 ± 13.9	0.327
DBP, mmHg	74.3 ± 11.3	74.2 ± 11.5	74.5 ± 11.1	0.227	75.0 ± 11.0	74.5 ± 11.3	0.143
Current smoker, *n* (%)	1001 (18.6%)	774 (22.9%)	227 (11.2%)	<0.001	334 (19.5%)	217 (12.6%)	<0.001
Exercise, *n* (%)	3320 (61.6%)	1897 (56.3%)	1423 (70.5%)	<0.001	994 (58.0%)	1200 (69.9%)	<0.001
Alcohol, *n* (%)	2494 (46.2%)	1719 (50.9%)	775 (38.4%)	<0.001	792 (46.2%)	711 (41.5%)	0.006
History of hypertension, *n* (%)	1157 (21.4%)	616 (18.2%)	541 (26.8%)	<0.001	432 (25.2%)	426 (24.8%)	0.844
History of DM, *n* (%)	700 (13.0%)	375 (11.1%)	325 (16.1%)	<0.001	254 (14.8%)	269 (15.7%)	0.506
History of dyslipidemia, n (%)	1143 (21.2%)	595 (17.6%)	548 (27.1%)	<0.001	403 (23.5%)	430 (25.1%)	0.301
History of CVD, *n* (%)	282 (5.2%)	156 (4.6%)	126 (6.2%)	0.012	101 (5.9%)	98 (5.7%)	0.884
History of cancer, *n* (%)	207 (3.8%)	103 (3.1%)	104 (5.2%)	<0.001	77 (4.5%)	74 (4.3%)	0.868
Glucose, mg/dL	101.4 ± 20.6	100.5 ± 21.3	102.9 ± 19.2	<0.001	102.9 ± 22.7	102.6 ± 19.1	0.642
Total Cholesterol, mg/dL	189.2 ± 38.9	190.7 ± 37.8	186.8 ± 40.6	<0.001	189.5 ± 39.5	186.9 ± 39.9	0.053
LDL, mg/dL	125.3 ± 37.4	126.5 ± 36.7	123.3 ± 38.6	0.003	125.5 ± 37.8	123.5 ± 38.1	0.127
HDL, mg/dL	57.8 ± 15.2	57.6 ± 15.3	58.1 ± 14.9	0.229	58.0 ± 15.4	57.7 ± 14.9	0.589
Triglyceride, mg/dL	118.1 ± 78.7	121.3 ± 81.6	112.8 ± 73.1	<0.001	119.0 ± 78.9	113.7 ± 75.3	0.046
TyG Index	8.5 ± 0.6	8.5 ± 0.6	8.5 ± 0.6	0.025	8.6 ± 0.6	8.5 ± 0.6	0.013
AIP	0.6 ± 0.7	0.6 ± 0.7	0.5 ± 0.7	<0.001	0.6 ± 0.7	0.6 ± 0.7	0.046
AST, IU/L	27.2 ± 13.4	26.5 ± 13.4	28.2 ± 13.5	<0.001	27.4 ± 13.4	27.8 ± 13.2	0.413
ALT, IU/L	25.7 ± 17.9	25.7 ± 18.4	25.8 ± 16.9	0.796	25.3 ± 17.4	25.9 ± 17.2	0.373
GGT, IU/L	32.7 ± 42.5	34.6 ± 45.9	29.6 ± 35.9	<0.001	33.3 ± 40.4	30.5 ± 37.7	0.034
CRP, mg/L	1.3 ± 3.0	1.3 ± 3.1	1.3 ± 2.7	0.574	1.4 ± 3.8	1.3 ± 2.9	0.594
SLD, *n* (%)	2605 (48.3%)	1614 (47.8%)	991 (49.1%)	0.379	897 (52.3%)	830 (48.4%)	0.024
MASLD, *n* (%)	2479 (45.9%)	1525 (45.2%)	954 (47.3%)	0.146	851 (49.6%)	798 (46.5%)	0.076
ALD, *n* (%)	109 (2.0%)	77 (2.3%)	32 (1.6%)	0.097	38 (2.2%)	28 (1.6%)	0.263
Framingham risk score	8.7 ± 8.0	8.3 ± 8.1	9.3 ± 8.0	<0.001	10.1 ± 9.0	9.3 ± 8.1	0.009

Data are expressed as mean ± standard deviation or n (percentage). Before matching, *p*-values were calculated using *t*-tests for continuous variables and chi-square tests for categorical variables. After matching, the *p*-values were calculated using the Gee method. Matching variables: age, sex, and 1:1 exact matching Abbreviations: SD, standard deviation; BMI, body mass index; SBP, systolic blood pressure; DBP, diastolic blood pressure; DM, diabetes mellitus; CVD, cardiovascular disease; LDL, low-density lipoprotein; HDL, high-density lipoprotein; TyG, triglyceride and glucose; AIP, atherogenic index of plasma; AST, aspartate aminotransferase; ALT, alanine aminotransferase; GGT, γ -glutamyl transferase; CRP, C-reactive protein; SLD, steatotic liver disease; MASLD, metabolic dysfunction-associated steatotic liver disease; ALD, alcohol-related liver disease.

**Table 2:** Multiple regression analyses of the highly adherent group to Mediterranean Diet Score with Metabolic Liver Conditions.

		SLD		MASLD		ALD	
		OR (95% CI)	*p*-Value	OR (95% CI)	*p*-Value	OR (95% CI)	*p*-Value
Model 1 ^a^	Less adherent group	Ref		Ref		Ref	
	Highly adherent group	0.842 (0.732–0.969)	0.017	0.871 (0.756–1.004)	0.056	0.686 (0.683–0.689)	<0.001
Model 2 ^b^	Less adherent group	Ref		Ref		Ref	
	Highly adherent group	0.834 (0.714–0.973)	0.021	0.856 (0.730–1.003)	0.055	0.330 (0.103–1.054)	0.061
Model 3 ^c^	Less adherent group	Ref		Ref		Ref	
	Highly adherent group	0.822 (0.703–0.960)	0.014	0.842 (0.717–0.989)	0.037	0.382 (0.111–1.317)	0.128
Model 4 ^d^	Less adherent group	Ref		Ref		Ref	
	Highly adherent group	0.818 (0.700–0.957)	0.012	0.839 (0.714–0.986)	0.033	0.677 (0.671–0.683)	<0.001

OR, odds ratio; CI, confidence interval; SLD, steatotic liver disease; MASLD, metabolic dysfunction-associated steatotic liver disease; ALD, alcohol-related liver disease. ^a^: Unadjusted model. ^b^: Adjusted for age, sex, and body mass index (BMI). ^c^: Adjusted for age, sex, BMI, alcohol intake, smoking status, and exercise. ^d^: Adjusted for age, sex, BMI, alcohol intake, smoking status, exercise, history of hypertension, diabetes, dyslipidemia, cardiovascular disease, and cancer. *p*-value by a generalized mixed model with a logit link.

## Data Availability

The original contributions presented in the study are included in the article, further inquiries can be directed to the corresponding author.
